# Sharing risk management: an implementation model for cardiovascular absolute risk assessment and management in Australian general practice

**DOI:** 10.1111/j.1742-1241.2008.01769.x

**Published:** 2008-06

**Authors:** Q Wan, M F Harris, N Zwar, S Vagholkar

**Affiliations:** 1 Centre for Primary Health Care and Equity, School of Public Health and Community Medicine, University of New South WalesSydney, NSW, Australia; 2 General Practice Unit, Fairfield HospitalFairfield, NSW, Australia

## Abstract

**Purpose:**

Despite considerable work in developing and validating cardiovascular absolute risk (CVAR) algorithms, there has been less work on models for their implementation in assessment and management. The aim of our study was to develop a model for a joint approach to its implementation based on an exploration of views of patients, general practitioners (GPs) and key informants (KIs).

**Methods:**

We conducted six focus group (three with GPs and three with patients) and nine KI interviews in Sydney. Thematic analysis was used with comparison to highlight the similarities and differences in perspectives of participants.

**Results:**

Conducting CVAR was seen as more acceptable for regular patients rather than new patients for whom GPs had to attract their interest and build rapport before doing so at the next visit. GPs’ interest and patients’ positive attitude in managing risk were important in implementing CVAR. Long consultations, good communication skills and having a trusting relationship helped overcome the barriers during the process. All the participants supported engaging patients to self-assess their risk before the consultation and sharing decision making with GPs during consultation. Involving practice staff to help with the patient self-assessment, follow-up and referral would be helpful in implementing CVAR assessment and management, but GPs, patients and practices may need more support for this to occur.

**Conclusions:**

Multiple strategies are required to promote the better use of CVAR in the extremely busy working environment of Australian general practice. An implementation model has been developed based on our findings and the Chronic Care Model. Further research needs to investigate the effectiveness of the proposed model.

**What's known**

Absolute risk (CVAR) acknowledging the multi-factorial causation of CVD has been recommended by many clinical guidelines. Despite considerable work in developing and validating CVAR algorithms, there has been less work for their implementation in clinical practice.

**What's new**

Multiple strategies are required to promote the use of CVAR in the busy working environment of Australian general practice. An implementation model has been developed based on our findings and the Chronic Care Model.

## Introduction

Cardiovascular disease (CVD) is the leading cause of death and disability in Australia (1). It is extremely costly to the healthcare system (2,3) and a large economic and social burden to individuals. There are a number of modifiable risk factors for CVD (4). In 2001, 53% of the Australian adult population had two or more of these risk factors (5). Primary prevention of CVD requires accurate assessment and effective management of its risk factors. Intervention programmes in general practice have proven successful in improving the quality of life in patients at high cardiovascular risk (6). Cardiovascular absolute risk (CVAR) is the probability of developing a cardiovascular event over a given time period (usually over 5 or 10 years). Because it acknowledges the multi-factorial causation of CVD, CVAR has been recommended worldwide by many clinical guidelines to tailor CVD primary prevention (7–12).

While there has been considerable work on developing and validating CVAR algorithms and tools (13), there has been little work on models for their implementation and evaluation of their impact on clinical care especially in Australian primary care. Previous qualitative research with general practitioners (GPs) identified some significant deficiencies and barriers to its use in general practice (e.g. deficiencies in computer records, inconsistency with regulations for prescribing and lack of patient understanding of CVAR concepts) (14). Paterson et al. (15) found that giving patients a simple risk assessment tool was effective in improving patent compliance in cardiovascular risk assessment process. This showed that interventions aiming at developing active participation and sharing of decision-making (SDM) (16) by patients could be a promising strategy. Moreover, the chronic care model (17) suggests that a combination of strategies is required to improve the quality of care and health outcomes. Therefore, in this study to develop a practical model for future implementation of CVAR in Australian general practice, we sought the opinions of patients, GPs and key informants (KIs) about CVAR assessment and management, patient self-assessment before the consultation and SDM in implementing CVAR.

## Methods

### Study sample and recruitment

Data collection was conducted from 2005 to 2006 in Sydney. Three Australian Divisions of General Practice consented to participate. GPs were recruited by invitation via Division newsletters. Patients were recruited through the participating GPs and from participants in group programmes run by the Divisions (e.g. diabetes education or physical activity programme, which helped us include patients with higher risk). Eligible patients were more than 40 years old, had at least one CV risk factor (smoker, overweight/obese, insufficient physical activity, hyperlipidaemia, hypertension, diabetes, family history of hypercholesterolaemia or first-degree relative who developed coronary heart disease before age 60) and had no previous/current CVD/stroke. Letters of invitation were mailed to the key health professionals from different organisations including GP academics involved in CVD research, nursing in general practice; non-government organisations involved in CVD, government policy makers, Division of General practice and consumer bodies. Ethical approval for the study was obtained from the Human Research Ethics Committee of the University of New South Wales. Participants gave full informed written consent.

### Data collection and analysis

Patient and GP focus groups (FGs) were conducted separately for approximately 2 h each. FGs and KI interviews were based on a semi-structured interview theme list (Table 1), developed from the literature review and our previous research (14,18). New Zealand CVAR electronic and paper-based calculators [recommended by Australian guidelines (7,9)] and an initial patient self-assessment form [developed by our research team based on some validated questions (19)] were also shown to participants to expand the discussion on themes 2 and 3. We continued to conduct FGs until no new information about the important themes emerged. KI interviews were conducted via telephone, each lasting about 40 min.

**Table 1:** Main theme guide

1. Views on CVD risk assessment: why, for whom, when, how
2. Views on CVAR assessment and management: (only for GPs and KIs) why, how, barriers/facilitators
3. Views on patient self-assessment and management of CVD risk: why. how, barriers/facilitators
4. Views on shared assessment and management of CVAR: why, how, what roles for patients, GPs and other health professionals

CVD, cardiovascular disease; CVAR, cardiovascular absolute risk; GPs, general practitioners; KIs, key informants.

Both FGs and KI interviews were audiotaped and transcribed. The accuracy of transcripts was checked prior to being transferred to qsr nvivo 7 software for analysis (20). The transcripts were analysed for themes taking into account study aims, group interactions, participants’ backgrounds and knowledge. A thematic coding frame was developed and consensus about the coding was reached through discussion among the research team. Transcripts were coded separately by two authors (QW and MH) and then checked for consistency. Where there were differences these were discussed to resolve them. Different ways of approaching the same subject confirms the validity of data and results in an increased understanding of complex phenomena. Therefore, comparison was used to highlight similarities and differences in the perspectives of the three groups (patients, GPs and KIs) (21). Analysis from all sources was discussed with all authors and the implementation model for CVAR emerged from these discussions, reflection on previous research (14,18) and integration into the chronic care model.

## Results

### Demographic information

In total, six FGs (three with GPs and three with patients) and nine KI interviews were conducted in this study. Twenty-two GPs participated in three GP groups (ranged from four to 13 participants, one FG in each division): They were aged more than 30 years with an average working experience of 25.7 years, seven were women and 14 were solo practitioners. Twenty-six patients participated in three patient groups (ranged from six to 10 participants, one FG in each division): They were aged between 42 and 81 years (mean: 63.5), 12 referred from GPs and 14 from patient group programmes, 15 were women and 12 had one or two major CVD risk factors and 14 had more than two. The nine KIs were from eight organisations: two Divisions of General Practice, the National Prescribing Service, the Department of Health and Aging, the National Heart Foundation of Australia, the Australian Division of General Practice, the Royal Australian College of General Practitioners and the Consumer researcher origination in Chronic Illness Alliance.

### Conducting CVAR assessment in a GP consultation

All GPs felt that CVAR assessment could help them to target those patients at greater risk and to spend less time with people who were at low risk.

“It depends on the level of risk. If the risk is relatively low you would give advice that's relevant to that aspect of risk, and if the risk is high you then you obviously have to think of what things to put in place immediately, down the track, follow up (GP group 1).”

Most GPs and KIs also felt that CVAR assessment could help them to prioritise each patient's risk, better allow them to tailor their management.

“I think the strength of the absolute risk concept is that it improves the targeting of certain interventions, so that you have a greater accuracy when you’re prescribing things like Statins but also a greater accuracy and confidence when you prescribe just behavioural measures like diet and exercise… (KI 6).”

For regular patients who presented opportunistically, most GPs in this study were of the view that they would explain the situation to patients and ask them to come back for a later assessment (if not urgent). Similarly, for new patients most GPs preferred to sow the seeds at the first visit, emphasise the importance of risk assessment and have patients come back. For young adults, some GPs commented that it might be more difficult for them to come back because of their occupational commitments. However, some GPs said that most of their patients would come back if they have arranged the consultation for them.

All GPs agreed that they needed to focus on the likely impacts on how patients functioned in their daily life rather than simply try to scare patients with the risk of sudden death. Meanwhile patients felt that they needed to take a positive attitude.

“It's not what the diagnosis is, it's that ‘I can't function, I can't look after the family, can't drive, and can't hold down a job – those are the important things (GP group 2).”

“So you have to be positive and you can still work and do exercise, and your golf, tennis or whatever, but I think a positive attitude is what you have to have, so you won't go down, and you will know more about it and learn more about it (patient group 3).”

Most GPs thought that patients liked a personal approach and if they talked to patients and showed that they were interested then they might get a better response from the patients with their plan to reduce their risk. This approach allowed GPs to tailor their approach in terms of the patients’ knowledge, attitudes and interest.

“You have to judge the people, at the time you have to make an informed decision as to how much information is going to sink in, what sort of response you’re going to get (GP group 1).”

Asking patients to change their usual lifestyle was difficult. GPs commented that it was important to build a rapport before starting to advise patients. It was even better if GPs could use patients’ own language and consider their cultural background.

Most GPs in this study agreed that 5 years was better to use than 10 years as a predictive period as the latter may be too long for patients to be able to relate to. They felt that the quantitative scoring of CVAR might be difficult for patients to understand. To interpret the CVAR results more clearly to patients, GPs said they needed more consultation time and clear and simple messages. This could not be simply addressed by giving all patients a piece of paper explaining their risk as explanation needed to be personalised for each patient. Some patients mentioned difficulty with technical words used by GPs, For example:

“Because he's called it CVD or cardiovascular disease, it's too much to myself to take in but it seems like – if he said, ‘you’ve probably got a heart problem’ then I haven't got a problem with that because that's easy to understand and easy (patient group 1).”

Time was a common barrier raised by patients, GPs and KIs. However, although most GPs in this study thought that time was a major concern especially where there were multiple risk factors, some of them were happy to book a long consultation to deal with this problem at a later visit if they considered it necessary, especially where there were large numbers of patients waiting. All GPs, KIs and patients agreed that computer programs would facilitate CVAR assessment during general practice consultations. This had the potential to save time providing they were easy to use.

### Patient self-assessment

Most patients interviewed were happy with the traditional model where GPs provided the advice and management. And they relied on their GPs to look after them, even while agreeing that patients should take more responsibility. However, some KIs and GPs questioned the traditional model – emphasising the central role of patients. All KIs felt that self-assessment was an opportunity for patients to look at their own risk behaviours and a trigger to help identify who may benefit from undertaking a fuller risk assessment by the GP. They believed that a self-assessment form could also benefit GPs by saving their time.

Although some GPs doubted the reliability of patient self-report of their smoking or alcohol consumption, all GPs agreed that self-assessment was helpful and could be used as a trigger to initiate discussion of risk factors. They also felt that it could help save their time and improve their understanding of patients’ risk.

“I imagine it acting like a springboard for discussion – the most important thing about people filling in a check list, and it isn't the number that pops out of the box, it's ‘oh, I see you’re a smoker, I see your father had a heart attack, tell me what happened there’ and exploring some of those, and ‘why do you keep smoking’– [It is] almost a springboard for discussion rather than a calculator for risk…it pops out? (GP group 2).”

Patients felt that a self-assessment could increase their awareness of their risk and help save the doctor's time.

“I'd been able to do a self-assessment some time, I would find that that would be helpful (patient group 1).”

Most GPs preferred their patients to complete the short self-assessment form in the waiting room rather than having it mailed to them or giving it to them to take home as they thought that patients probably would lose or not complete it. All KIs supported the usefulness of the self-assessment in the waiting room as patient were preoccupied thinking about their health at this time. Furthermore, most patients in this study expressed that patients were happy to fill in the form in the waiting room.

“Actually you'd be happy to do anything when you’re waiting in the doctor's (patient group 2).”

### Shared approach in CVAR assessment and management

In general, all the participants (patients, GPs and KIs) agreed that a shared approach among patients, GPs and other health professionals (e.g. diabetes educator, dietician, pharmacist, physiotherapist and other practice staff, etc.) would be a promising way to help improve the rate of CV risk assessment and management. From the most GPs’ points of view, this shared approach would provide an opportunity for them to share their large workload with other practice staff (receptionists, practice nurse or practice manager) who could help prompting and assisting patients to do the self-assessment, providing information and education, arranging referral and follow. However, one solo practitioner said:

“Each practice is different, in a solo practice they (other practice staff) probably don't have time to get involved… (GP group 1).”

Both KIs and GPs endorsed the idea that practice nurses could help in the assessment. However, many GPs stated that they could not afford to employ a practice nurse unless there was some government financial support.

All GPs thought engaging patients in shared care could make patients more responsible for their own health. Most patients commented they could benefit from increased awareness by conducting the self-assessment before the consultation (the first step in this shared approach) and discussing more with GPs during the consultation. Also they felt that they could benefit from other health professionals’ involvement in assessing and managing their risk.

However, some KIs were concerned that the shared approach and need for co-operation among patients, doctors and other practice staff (nurse and/or receptionist) might increase the complexity of management tasks as involving other practice staff necessitated training and funding for their time. Some GPs worried that current rules and regulations limited the implementation of this shared approach. Some patients questioned whether advice from other practice staff would be sufficiently consistent:

“They differ, one person told me not to touch milk, the dietitian said have at least two a day, one thing and the dietitian told me something else, to have eight slices of bread, grain bread… (patient group 3).”

Given the current heavy workload of GPs in Australia KIs were unanimous that general practice needed more infrastructure and training support to undertake CVAR assessment and management properly.

“Really it's a team approach. It's very important to do these sorts of things systematically, so the whole practice is set up with the right practice management systems in place. A practice manager or other in the practice needs to have responsibility of co-ordinating a systematic and team approach. As mentioned elsewhere, practice nurses may have a key role (KI interview 6).”

General practitioner, patients and KIs all agreed that it was important to increase patients’ awareness of cardiovascular risk through public campaigns and messages in the media. However, there were mixed opinions among GPs about the usefulness of written information for patients to take home. In contrast, patients welcomed written information for them to take home. They suggested that this may need to be given at a follow-up consultation.

“I don't think that the third point, take home the results would happen straight away because mostly they [patients] might have to go off and have a blood test or some other test, so it would be in the follow up appointment…because by then they might know the cholesterol or other factors that come in from the test… (patient group 1).”

## Discussion

Cardiovascular absolute risk assessment was more acceptable for patients who were longer-term patients of the GPs. A relationship and trust may need to be established prior to attempting CVAR assessment and recommending changes to patients’ lifestyles. This suggests that risk assessment and preventive programmes such as the CVAR assessment need to be seen within the context of an ongoing relationship with the patient's usual GP rather than as an opportunistic activity.

The CVAR has been recommended to assess the total risk of CVD in general practice by many clinical management guidelines in Australia (7,22,23). As was acknowledged by KIs and GPs in this study, the uptake has been very limited so far. However, it was encouraging that GPs participating in this study all had positive attitudes towards CVAR, believing that it would help them to target patients with greater risk and tailor their management more effectively. Despite these positive attitudes, our previous research has indicated that many diabetic patients with high CVAR were not receiving pharmacological interventions (18). This suggests that the barriers may not be simply attitudinal and that the problem may be in the translation of these intentions into practice.

Most GPs felt explaining CVAR to patients would be difficult and time consuming. Multiple strategies were required to support this including communication, training and financial support. Those GPs expressed a need for the terminology to be simplified. Medical Jargon was disliked by all patients as well. According to most GPs, patients needed to be able to relate the explanation of CVAR to the likely impact on their own lives. This could not be addressed by simply providing patients with educational material. Long consultations and a follow-up consultation were often necessary to deal with the time problem in the busy work environment of Australian general practice. In Australia, for patients with complex chronic illness, care plans provide a useful vehicle for this (24). However, for other patients who do not yet have a chronic illness and are not eligible for care plans, providers may feel the reimbursement is inadequate for the time involved in the long CVAR assessment.

Policy makers have been keen to promote patient self-management because of its cost-effectiveness especially in chronic disease (25–28). In this study, patient self-assessment of CVAR was seen as facilitating self-management of risk. Patients, GPs and KIs were positive about patient self-assessment especially if this was conducted in the waiting room immediately prior to seeing the GP. Although there are other possible ways in which such information might be collected (including from the electronic record), self-assessment was seen as a useful way of increasing patient awareness and engagement. This was consistent with other research (15,29).

Shared decision making is being advocated as a useful model to better engage patients and other health professions in the clinical care (16). In this study, sharing risk assessment and decision making by patients and GPs prior to and during the CVAR consultation was supported by all the participants (patients, GPs and KIs) to increase patients’ responsibility by helping them to understand what to do and why. Patient engagement before the consultation and more engagement during the consultation would help patients to develop their self-management skills, which could improve health outcomes. Sharing roles with other practice staff was also supported by all the participants to improve GPs’ management of CVAR. These roles included prompting and assisting patients to do the self-assessment, providing information and education, arranging referral and follow up.

However, sharing the task of CVAR assessment among patients, GPs and other health professionals needs to be supported by effective systems and arrangements (including guidelines, communication and information systems). Funding may be needed to make them possible. Recently, the Australian government has introduced a number of new Medicare items for health checks and preventive in adults (30). These provide an important opportunity for assessment of CV risk in patients who do not yet have a chronic disease. Obviously practices still need to have the capacity to carry these out – something that will be challenging for solo practices without practice nurses or allied health staff.

**Figure 1:**
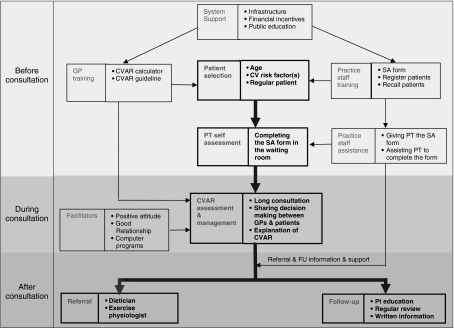
An implementation model of cardiovascular absolute risk in Australian general practice. GP, general practitioner; PT, patient; CVAR, cardiovascular absolute risk; SA, self-assessment; FU, follow-up

In summary, based on our research findings and its integration into the chronic care model, self-management and SDM theories, we have developed a shared implementation model of CVAR assessment and management (Figure 1) in which patients self-assess their CVD risk factors prior to the consultation, are educated in their own risk, and engage with GPs and other health professionals in decision making about the assessment and management of CVAR. This model may be a systematic combination of multiple strategies that is required for implementation of CVAR in Australian general practice.

Even though the sample of our FGs was restricted to Sydney area it included GPs from three Divisions of General Practice, and patients from both general practices and diabetes and physical activity programmes in those three Divisions of General Practice. This sample together with KIs from different organisations in Australia generated a diverse range of opinions. Of course this qualitative study was unable to evaluate the effectiveness of the model. However, it does provide evidence and direction for further research and policy initiatives designed to promote the better use of CVAR in Australian general practice.
